# Medical students’ knowledge on cancer predisposition syndromes and attitude toward eHealth

**DOI:** 10.1007/s00404-023-07266-4

**Published:** 2023-11-07

**Authors:** Juliane Nees, Farina Struewe, Sarah Schott

**Affiliations:** 1 Department of Gynecology and Obstetrics, University Hospital Heidelberg, Im Neuenheimer Feld 440, 69120 Heidelberg, Germany; 2 Department of Pediatric Hematology and Oncology, Hannover Medical School, Hannover, Germany

**Keywords:** Cancer predisposition syndrome, Medical education, eHealth, Li-Fraumeni Syndrome, Precise medicine, Rare disease

## Abstract

**Purpose:**

Individuals with cancer predisposition syndromes (CPS) inherit elevated cancer risks. Medical supply gaps for people at risk of CPS cause insufficient outreach and miss potential benefits of individualized care strategies. Increased awareness of CPS and progress in the eHealth sector are untapped sources of health care improvement for affected individuals.

**Methods and results:**

This study addressed German–speaking medical students with an online questionnaire in respect to their knowledge of CPS, their medical education, and perspectives. The study population (*n* = 404) reported interest in and knowledge of CPS, supported by a satisfactory and sustainable education for their prospective patient care. The next generation of doctors would implement eHealth to improve medical services. Skepticism about digitization was claimed by students. They were especially concerned about deterioration in the physician–patient relationship, data abuse, dependence on technology, and incorrect diagnoses.

**Conclusion:**

Due to increasing diagnosing of CPS and deeper knowledge, this topic is essential for the curriculum in medical schools. In particular, care providers need know-how on identifying patients at risk for a CPS, certain diagnostic and therapeutic steps, surveillance and prophylactic strategies to improve patients’ outcomes. Education in medical school as well as implemented eHealth seems to have potential to meet this demand in an upcoming era of personalized medicine.

What does this study add to the clinical work.

Medical teaching on cancer predisposition syndromes should be expanded to improve knowledge and individualized and personalized healthcare.

**Supplementary Information:**

The online version contains supplementary material available at 10.1007/s00404-023-07266-4.

## What does this study add to the clinical work.

**Table:** 

Medical teaching on cancer predisposition syndromes should be expanded to improve knowledge and individualized and personalized healthcare.	

## Introduction

Individuals with cancer predisposition syndromes (CPS) inherit elevated cancer risks and their care demands specific medical strategies. In times of increasingly personalized medicine, the genome of patients plays an ever-increasing role in the choice of diagnostic and therapeutic strategies [1, 2]. For several CPS, complex surveillance has been established. In terms of Li-Fraumeni Syndrome (LFS), one of the most penetrant CPS, adherent behavior concerning surveillance can achieve an overall survival benefit [2–5], although broad clinical implementation worldwide is lacking, yet. Specific breast cancer surveillance strategies including breast MRI that were successfully implemented within the German consortium for hereditary breast and ovarian cancer led to benefits for high-risk BRCA carriers [6]. Therapeutically, specific treatments are required for CPS carriers to avoid over- and undertreatment or even toxic regimens [7, 8]. More precise strategies, e.g., PARP inhibitors, for carriers of (likely) pathogenic somatic or germline BRCA*,* have been approved and require early identification of people who could potentially benefit.

On the other hand, the rapid gain in knowledge on specific CPS variants demands fast, evidence-based translation into clinical practice [9]. Knowledge transfer, outstanding research communication, and continuous education for care providers besides specific CPS clinics are crucial for outreach and clinical implementation.

Today, an underachievement of clinical translation is noted in the literature [10]. Medical supply gaps with a lack of identified individuals at risk for a CPS cause insufficient outreach with missed potential benefits from genetic testing and cancer preventive or individualized care strategies [10–12]. Additionally, affected individuals had noted physicians’ lack of CPS knowledge and often felt like an educator rather than a patient [13].

The digital eHealth sector has become an integral part of the medical field with untapped potential to improve care on even more patient-centric level [14]. During the COVID pandemic, it proved to be a safer route to reach patients, especially for those who otherwise would have missed medical contact [15]. However, its future implementation and usage will be driven by medical professionals’ as well as patients´ uptake. Its potential for CPS carriers and the extent of supply gaps has not yet been investigated.

Here, we questioned the future generation of physicians—German-speaking medical students—on their knowledge about CPS and eHealth as well as their strategies for gaining knowledge. The captured status quo shall guide aspects of medical education and patient care along modern supply structures.

## Methods

### Survey design

The online survey was designed in an interactive manner by physicians from different medical specialties and universities in Germany. The design resulted in a structured format: single-, multiple-choice, and free-text questions were included with answer types assigned to nominal, ordinal, and ratio scales. In total, 41 items were captured: (a) personal data as well as current educational status (*n* = 19), (b) knowledge and attitude toward CPS (*n* = 11), and (c) attitudes towards eHealth and the digitalization of medicine in general and in relation to CPS and personalized medicine (*n* = 11). (Supplementary Files) There was no time limit in which the questions had to be answered. Participation was possible with any device with internet access to Survey Monkey. All participants provided online consent prior to study start as access to the survey was not given without consent. The study was approved by the ethical committee of the Medizinische Hochschule Hannover (number 10015_BO_K_2021).

### Study population

Study participants were recruited via lectures and posters, student mailing lists, university newsletters, and social media channels. German-speaking medical students ≥ 18 years old at any level of education were eligible to participate in this anonymous online survey using Survey Monkey (www.surveymonkey.com) between October and December 2021.

### Statistical analysis

Statistical analyses were performed with Microsoft Excel Professional Plus 2019. The quantitative data were evaluated using descriptive analyses (frequencies and mean values). Percentages relate to the number of participants who answered the relevant question. Empirical distributions were determined by absolute and relative frequencies for categorical variables. The results are to be considered descriptive without confirmatory value because all participants included met the inclusion criteria.

## Results

### Study cohort, study experiences, and future perspectives

The study cohort comprised 404 students: 293 females (77.51%), 84 males (22.22%), and 1 diverse gender (0.26%). The majority studied in Germany (*n* = 351, 92.86%; Switzerland *n* = 15, 3.97%, 7 in Austria: *n* = 7, 1.85%, 5 in Hungary: 1.32%). The average participant was aged 24 (median; range 18–41) and currently in a clinical semester (*n* = 234, 61.91%; 89 students attended a preclinical semester (23.55%) and 55 practical year (14.55%)). Half of the study population had experienced clinical training (*n* = 209, 57.10%) mostly in the mandatory general medicine internship (*n* = 152, 73.08%), followed by internal medicine (*n* = 90, 43.27%), surgery (*n* = 59, 28.37%), anesthesiology (*n* = 46, 22.12%), gynecology or pediatrics (each *n* = 39, 18.75%).

Overall satisfaction with their training (*n* = 341, 92.16%) and good preparation for their future medical career (*n* = 288, 77.84%) was reported. Teaching formats such as bedside teaching (*n* = 283, 76.49%), seminars (*n* = 241, 65.14%), skills labs (*n* = 240, 64.86%), and simulator training (*n* = 192, 51.89%) supported their assessment and ranked most advantageous.

Research involvement was rather low (no research: *n* = 240, 64.86% vs research: *n* = 130), but some were focusing on their doctoral thesis (*n* = 108, 26.73%) or employment as a student assistant within a research group (*n* = 40, 9.9%). Prospectively, half the study population intended to complete a doctoral thesis along with medical school (*n* = 185, 50.55%). Future preferences for residency were mainly missing. Those with a preference favored internal medicine at a university hospital (*n* = 114, 30.16%) or private practice (*n* = 80, 21.16%), followed by surgery, pediatrics and gynecology/obstetrics. A clinician scientist career (*n* = 140, 38.25%) was an option for a third after medical school.

### Students’ knowledge on cancer predisposition syndromes

**Fig. 1:**
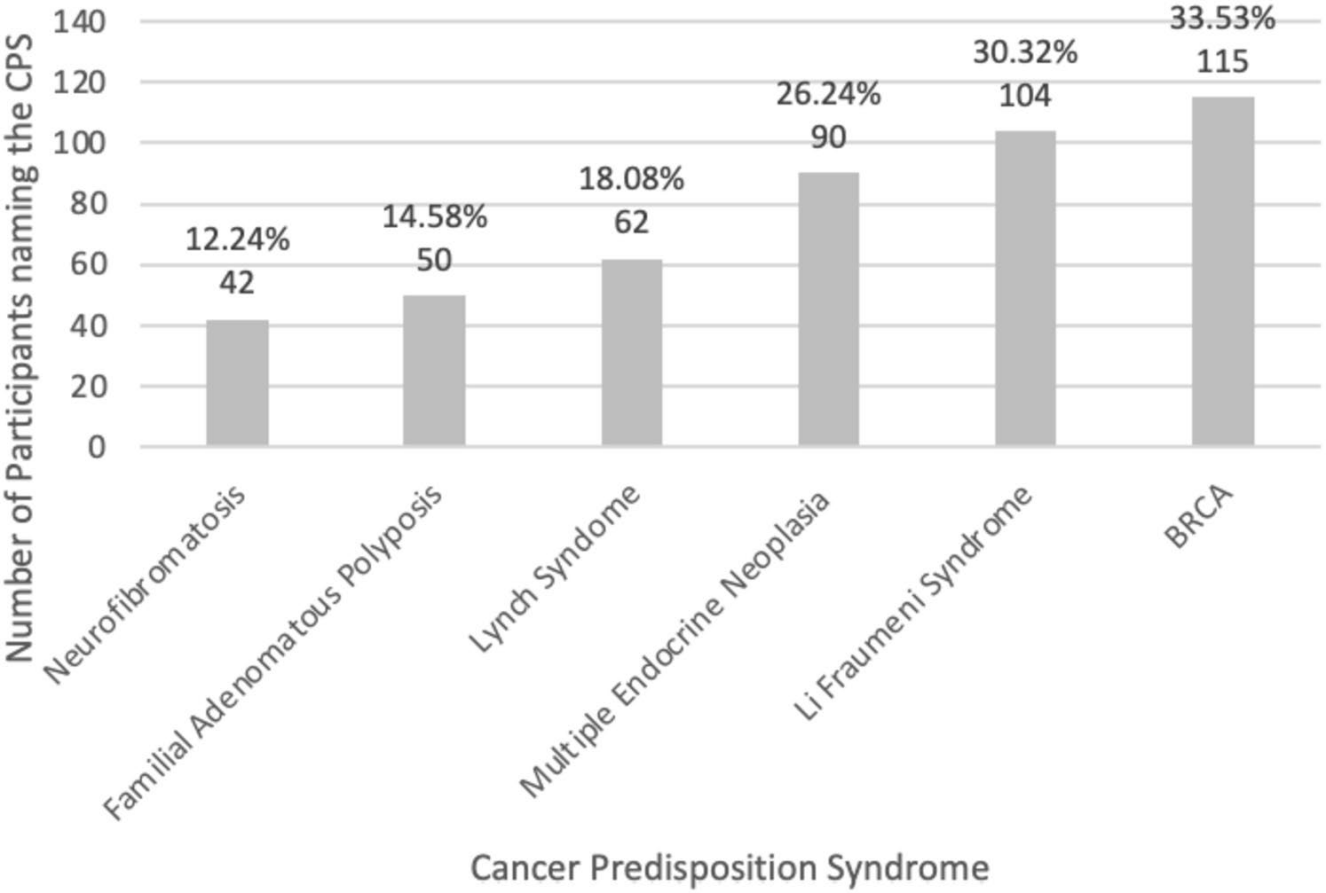
Best known cancer predisposition syndromes-among the study cohort

**Fig. 2:**
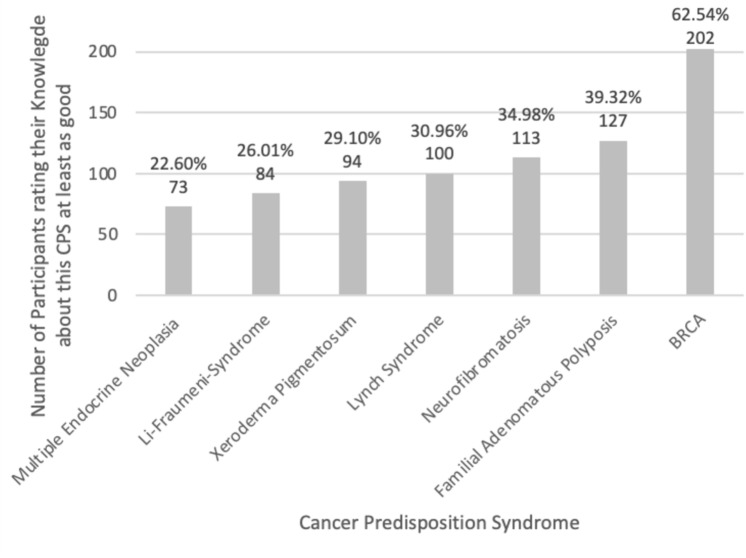
Number of students rating their knowledge of specific cancer predisposition syndrome at least as good

Most study participants (*n* = 279, 77.07%) had not met a person with CPS throughout their career and rated their theoretical knowledge of CPSs as typical to that of their fellow students (*n* = 226, 69.97%). The most common CPSs of children or adults (BRCA, Li-Fraumeni-syndrome, multiple endocrine neoplasia, and Lynch syndrome) were the best known CPSs (Fig. 1); however, there was high variability among the study participants. Figure 2 shows the percentage of participants who reported very good or good knowledge of the corresponding CPS.

A majority of the study participants (*n* = 240, 74.31%) claimed awareness about CPS-specific surveillance programs (*n* = 240, 74.31%) and therapeutic recommendations (*n* = 231, 71.51%). However, more than half (*n* = 192, 59.44%) were not familiar that some standard therapies and diagnostics for people with CPS can be harmful. Interestingly, most students claimed to be interested in caring for cancer patients (*n* = 286, 79%) and people with CPS (*n* = 281, 77.63%), and did agree that knowledge of CPS is important to very important (*n* = 279, 86.37%) for their professional future. They stated at least a high demand for knowledge in this regard (*n* = 219, 67.80%). Many (*n* = 294, 91.02%) consider personalized medicine to be a useful addition to standard therapy and have the impression that medicine is becoming more and more complex (*n* = 317, 98.17%).

### eHealth

**Fig. 3:**
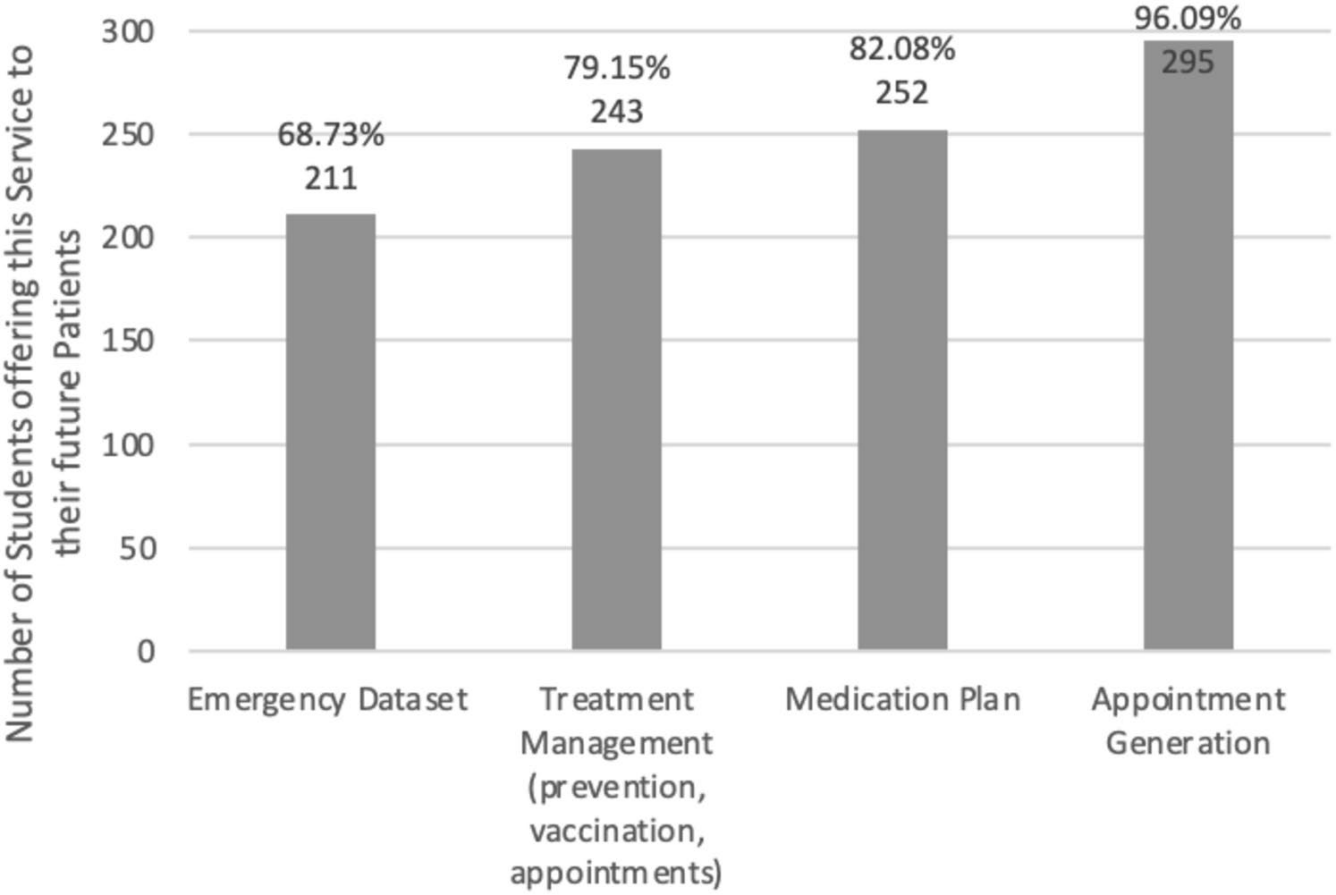
Students´ preferred services in a medical app they would recommend to their patients

**Fig. 4:**
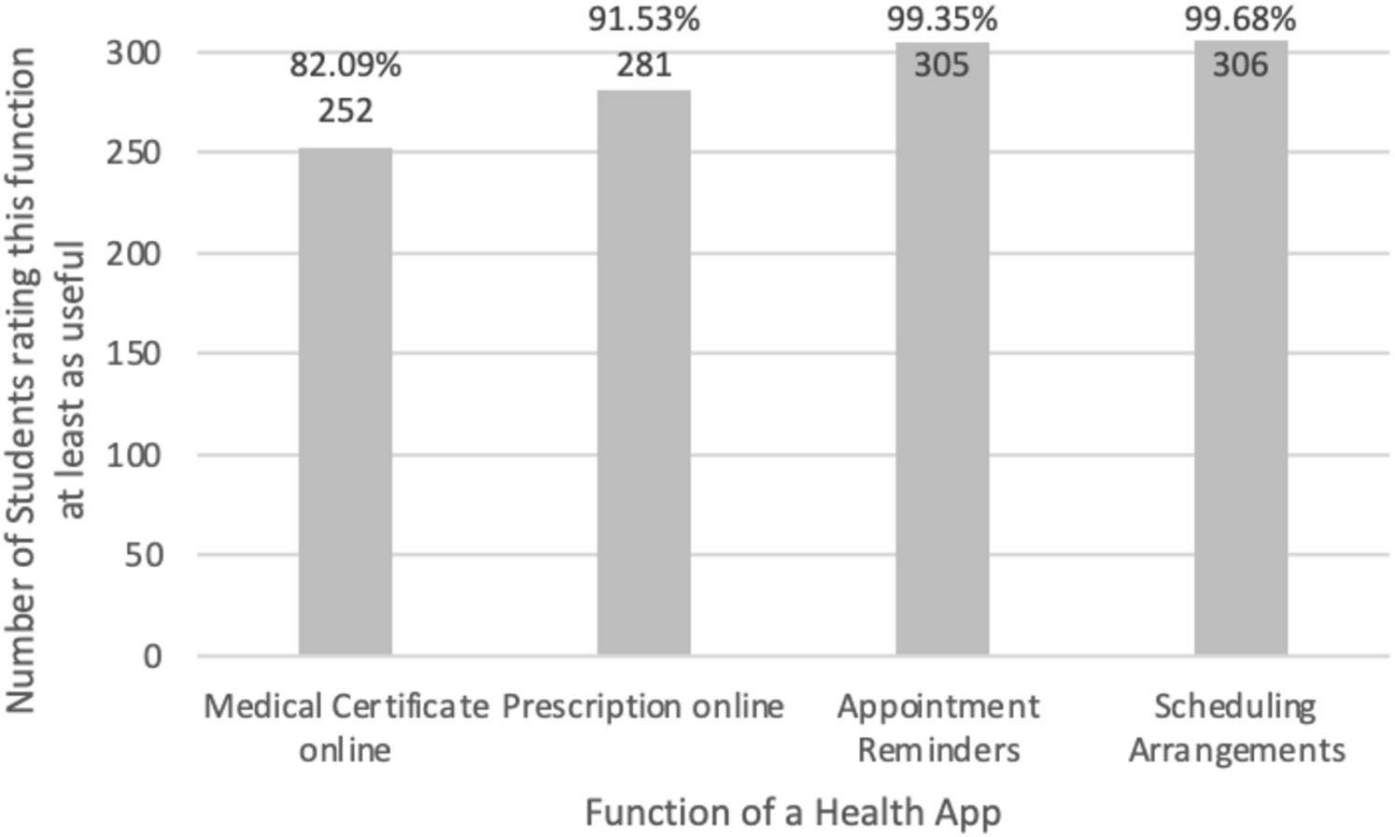
Frequency of students rating selected health app functions as useful or very useful

eHealth and its implementation in patient care revealed that several apps were already widely used. “Amboss”, a learning and reference app and website very frequently used for studies prior to the final exam, was known by 82 students (33.12%) and used by 75 (18.56%). Thirty-eight (9.40%) students were familiar with a COVID app and 21 (5.19%) use such an app. The corresponding preinstalled apps from smartphone manufacturers such as Apple and Samsung were grouped under “Health” and were known by 35 students (8.66%) and 21 (5.19%) used it. Furthermore, some study participants (*n* = 28, 6.93%) named an app offered by a health insurance company and 15 (3.71%) worked with it. 116 (28.71%) students didn’t know any medical apps, and 143 (35.40%) did not use a single medical app. Participants wanted the services noted in Fig. 3 for digital applications in patient care. Figure 4 shows the features rated most useful in a health app.

The potential improvement for CPS care and prevention by health apps was seen by the majority (*n* = 298, 97.07%) and would be recommended to patients by 294 students (95.77%).

Most of the students (*n* = 243, 79.15%) were of the opinion that the COVID pandemic has changed the way we deal with digitization in healthcare, with a great potential for the health care sector to improve. The students felt hopeful (*n* = 143, 48.64%) about the increasing digitization. Especially for data availability (*n* = 282, 95.92%), knowledge access (*n* = 277, 94.22%), data acquisition (*n* = 267, 90.82%), and quality (*n* = 247, 84.01%).

Skepticism (*n* = 138, 46.94%) about increasing digitization was shown by students who expressed concern about deterioration in the physician–patient relationship (*n* = 235, 79.93%), data abuse (*n* = 234, 79.59%), dependence on technology (*n* = 182, 61.90%), and incorrect diagnoses (*n* = 171, 58.16%).

## Discussion

The German-speaking medical students reported an interest in and basic knowledge of CPS supported by a satisfactory and sustainable education for their prospective patient care. The next generation of doctors would implement eHealth with a potential to improve medical services, especially for people with CPS.

Consciousness and fundamental knowledge on genetics has become increasingly important for any physician—not only in oncology [16]. However, to date, studies on genetic testing and patient referral for testing have shown that even in multidisciplinary centers, not everyone with a medical indication receives genetic referral [17]. Reasons for this lack may be physician knowledge gaps [18] to identify eligible people. To meet this need, standardized questionnaires as established in pediatric oncology [19] or algorithms [20] should be integrated in every oncological workup. eHealth decision support tools might open options to improve the rate. Other possibilities are patient-derived approaches, i.e., such as itrunsinmyfamily.com, that enables lay people to check their personal CPS risk and consult specialists themselves.

Awareness and the variety of approaches, including patient-derived care, should be part of medical school curriculums as well as continuing medical education for visibility and patient´s acceptance. Knowledge of appropriate surveillance, prevention options, e.g. prophylactic surgery or medication, by all physicians can be crucial for people with CPS. CPS-related tumors require precise diagnostic and therapeutic strategies in order to avoid harm from current standards of care, such as radiation or X-ray-based diagnostics that can be a risk for life-threatening secondary malignancies [21]. Even surgical material can cause health problems; for example, textured implants were discussed along with breast implant-associated anaplastic large cell lymphoma. In women with LFS [22], it might increase cancer development and needs further investigation. Dedicated centers for people with CPS may be one possibility to improve precise care through individualized surveillance including diagnostic and therapy. However, limited resources [20] and long distance to the nearest genetic clinic contribute to a lack of clinical routine for people at risk of a CPS [23].

There is a need for better awareness for CPS, so far almost 60% of the study participants were not familiar with such syndromes. (Virtual) bedside teaching or the integration of affected individuals into seminars, as the two most popular teaching formats stated by our cohort, are options to fill this gap of personal experience and access needs of people with a rare disease. We gained an extremely positive experience with LFS carriers sharing their story in a seminar with medical students. This might be a promising teaching model for outpatient “bedside” teaching, besides the educational tools in clinical setting. Therefore, it might be helpful to include digital patient cases to gain awareness of CPS. Modern approaches with promising results aiming to improving knowledge about human papillomavirusamong medical and dental trainees in the USA included interactive, web-based genetics curriculums [18], or online educational intervention [24]. In addition to the e-format of teaching modules, our study participants also see great potential in the use of health apps for their future patients, including people with a CPS. Since our preliminary study showed that the acceptance or use of a health app is not very high for LFS carriers (under review), the students (but also physicians) should not only be taught how to strengthen the compliance of patients regarding an app. Rather, information and education on digitization should also be integrated into medical school. In contrast to ours and other studies, where maintaining a good doctor–patient relationship and data protection are seen as the greatest danger of eHealth [25], it has been shown that the targeted use of eTools can even improve doctor–patient communication [26]. Furthermore, eHealth may meet the need to reach areas without sufficient medical supply via televisits and remote patient monitoring [27] and so improve CPS patients´ frustration of limited access [28]. Although apps are in use, there is an option to increase the spread of medical apps among medical students. One way of improving this is the project “DiKoMed” at the Duisburg-Essen medical school, which accompanies the students throughout their studies and introduces them to suitable eHealth possibilities adapted to the respective semester (29).

Personalized medicine, which is based on (epi)-genetic analyses and will play an important role especially for people with CPS, includes predicting which patients will likely respond to specific cancer therapies while avoiding unnecessary, ineffective therapies. The outlook of personalized medicine illustrates the importance of genetic analysis for therapy, prophylaxis, surveillance, and aftercare needing further investigation. eHealth or apps as a therapy diary or monitoring may also be a part of future care, with a need for prospective studies specially to meet the pitfalls of complex individualized treatments. Furthermore, the promising results of molecular tumor boards open options for better care. However, this also entails a need for motivated future doctors in the driver’s seat to support findings with (clinical) research.

### Supplementary Information

Below is the link to the electronic supplementary material.

Supplementary file1 (PDF 183 KB)

## Data Availability

Data available on request from the authors.
