# Supplementary material for: Medical students’ knowledge on cancer predisposition syndromes and attitude toward eHealth
Source: Arch Gynecol Obstet. 2023 Nov 7;309(4):1535–41. doi: 10.1007/s00404-023-07266-4 (PMC10894105; doi:10.1007/s00404-023-07266-4)
Supplement: Supplementary file 1 — Supplementary file1 (PDF 183 KB) [file 404_2023_7266_MOESM1_ESM.pdf]

Questionnaire

<u>Questions related to the person</u>
<u>Demographics</u>

1. Your gender?

   - Male
   - Female
   - Diverse

2. How old are you?

   - Free text

3. In which semester are you currently in?

   - 1
   - 2
   - 3
   - 4
   - 5
   - 6
   - 7
   - 8
   - 9
   - 10
   - Practical year

4. In which country are you studying in?

   - Germany
   - Switzerland
   - Austria
   - Other: free text

5. What specialty do you aim for?

   - Occupational medicine
   - Biochemistry
   - Otorhinolaryngology
   - Human Genetics
   - Hygiene and Environmental Medicine
   - Public Health
   - Pharmacology
   - Phoniatrics and Pediatric Audiology
   - Physiology
   - Radiotherapy
   - Transfusion Medicine

- Anatomy
- Microbiology, Virology and Infectious Disease Epidemiology
- Pathology
- Rehabilitation Medicine
- Laboratory Medicine
- Nuclear Medicine
- Forensic Medicine
- Urology
- Oral and Maxillofacial Surgery
- Neurosurgery
- Psychosomatics
- Radiology
- Ophthalmology
- Pediatric Psychiatry
- Psychiatry
- Dermatology and Venereal Diseases
- Neurology
- General Medicine (family doctor)
- Anesthesiology
- Gynecology and Obstetrics
- Pediatrics
- Surgery
- Internal Medicine
- No specialist aspired
- Not decided

6. What job are you aiming for after graduation (note: there are different definitions between the federal states)?

    - I don’t want to work in clinics
    - Medical care center
    - Hospital for primary care
    - Hospital for basic/standard care
    - Maximum care hospital (not a university hospital)
    - Medical practice
    - University Hospital
    - Not decided
    - Other (please specify), free text

<u>Medical education / Curriculum</u>

7. How satisfied are you with your curriculum?

    - Very satisfied
    - Rather satisfied
    - Rather dissatisfied
    - Very dissatisfied

8. In your opinion, are you well prepared by your curriculum for your professional life as a physician?

   - Yes, absolutely
   - Yes, mostly
   - No, rather not
   - No, not at all

9. Which training and development tools are particularly beneficial for you personally? Please select up to four of the answers that apply most to you!

   - Online lectures on the internet
   - Congress visits
   - External training
   - Formative intermediate exams
   - Lectures
   - Problem-oriented learning / Virtual patients
   - Simulator training
   - Skills lab
   - Seminars
   - Bed side teaching
   - Other (please specify), free text

10. Are you currently working scientifically?

   - Yes
   - No

11. If yes, for what purpose are you working scientifically?

   - Dissertation
   - Research assistant
   - Other (please specify), free text

12. Do you want to work scientifically in future?

   - Yes
   - No
   - I don't know, yet

13. Have you already finished a dissertation?

   - Yes, I have finished a dissertation
   - No, I am currently working on my dissertation
   - No, but I am planning to work on a dissertation during my studies
   - No, I want to do a doctorate after my studies
   - No, I don't want to do a doctorate

14. Have you already done medical traineeships?

- Yes
- No

15. In which specialties have you done your medical traineeships? (Multiple choice)

- Occupational medicine
- Biochemistry
- Otorhinolaryngology
- Human Genetics
- Hygiene and Environmental Medicine
- Public Health
- Pharmacology
- Phoniatrics and Pediatric Audiology
- Physiology
- Radiotherapy
- Transfusion Medicine
- Anatomy
- Microbiology, Virology and Infectious Disease Epidemiology
- Pathology
- Rehabilitation Medicine
- Laboratory Medicine
- Nuclear Medicine
- Forensic Medicine
- Urology
- Oral and Maxillofacial Surgery
- Neurosurgery
- Psychosomatics
- Radiology
- Ophthalmology
- Pediatric Psychiatry
- Psychiatry
- Dermatology and Venereal Diseases
- Neurology
- General Medicine (family doctor)
- Anesthesiology
- Gynecology and Obstetrics
- Pediatrics
- Surgery
- Internal Medicine

16. Have you experienced any patients with a cancer predisposition syndrome (CPS) during your internships / bed side trainings?

- Yes, several times
- Yes, once
- No, not yet

17. Are you interested in the care of oncology patients?

- Yes, very interested
- Yes, little interested
- No, rather less interested
- Not, not at all

18. Are you interested in the care of patients with genetic diseases?

    - Yes, very interested
    - Yes, little interested
    - No, rather less interested
    - Not, not at all

<u>Knowledge and attitude towards cancer predisposition syndromes (CPS)</u>

Cancer predisposition syndromes (CPS) are genetic variants that go along with a significantly increased risk of one or more cancer diseases during lifetime of affected individuals.

19. Please, note all CPS that come to your mind spontaneously. If you don't know any, just note "none".

    - Free text

20. How familiar are you with the following CPS?

	I am very familiar with this CPS	I am quite familiar with this CPS	I am not very familiar with this CPS	I have never heard of this CPS
BRCA	○	○	○	○
FAP	○	○	○	○
Neurofibromatosis	○	○	○	○
Xeroderma Pigmentosum	○	○	○	○
Lynch Syndrome	○	○	○	○
Li-Fraumeni Syndrome	○	○	○	○
Multiple Endocrine Neoplasia	○	○	○	○
Tuberous Sclerosis	○	○	○	○
Constitutional Mismatch Repair Deficiency	○	○	○	○
Noonan Syndrome	○	○	○	○
Peutz-Jeghers Syndrome	○	○	○	○
Ataxia Teleangiectasia	○	○	○	○
DICER1 Syndrome	○	○	○	○

21. Did you know that there are special surveillance recommendations for most of the CPS?

- Yes and I know what they are about
- Yes, but I don’t know what they are about
- No

22. Did you know that there are special therapy recommendations for most of the CPS in case of a cancer disease?

- Yes and I know what they are about
- Yes, but I don’t know what they are about
- No

23. Did you know that for some CPS some of the standard therapies and examinations can be harmful?

- Yes and I know which therapies/examinations should be avoided
- Yes, but I don’t know which therapies/examinations should be avoided
- No

24. Have you ever used the CPS atlas on the website http://www.krebs-praedisposition.de?

- Yes
- No, but I know about it
- No and I don’t know about it

25. How would you evaluate your knowledge about CPS compared to your fellow students?

- Above average
- Average
- Below average

26. In your opinion, what importance will the knowledge about CPS have for your future as a physician?

- Very important
- Important
- Not much important
- Not important at all

27. What claim do you have on knowledge about CPS for yourself?

- Very high
- High
- Low
- None
- I am not interested in this field

For individuals with a diagnosed CPS, it is important that therapies are planned individually with taking the underlying CPS into consideration. In this context, following questions are of interest:

28. How is your attitude towards personalized medicine?

- o In my opinion, it is a sensible addition to standard therapies
- o In my opinion, it is a sensible as the main therapy
- o In my opinion, it is only sensible in research so far
- o In my opinion, it is not sensible

29. Do you agree with the statement that medicine is becoming more complex?

- o Yes
- o No

<u>Knowledge and attitude towards eHealth</u>

E-Health as a link between medical informatics, public health and economics. It is becoming more important and has its use in the field of health services and information that are supplied via and improved by the internet and further technologies (Eysenbach 1999).

30. Which Apps in the field of health and medical care do you know (please note max. 5)? If you don't know any, note "none".

- o Free text

31. Which of these Apps do you use yourself? If you don't use any, note "none".

- o Free text

32. For what purpose would you offer digital supply for patients? (Multiple choice)

- o App as part of the therapy
- o Video consultation
- o Follow ups
- o Assessment of medical history
- o Information sheets
- o Access to patient documentation
- o Remote access to medical parameters (blood pressure, blood sugar, other)
- o Emergency dataset
- o Treatment management (prevention, vaccination, appointments)
- o Medication plan
- o Appointment scheduling
- o None
- o Other (please specify), free text

33. Please evaluate the following online functions of a health care App concerning their usefulness in everyday life.

- Psychological care via email
- Psychological consultation via phone
- Psychological consultation via video call
- Genetic consultation via phone
- Phone consultation
- Communication of medical findings / test results
- Medical certificate online
- Prescription online
- Health tracking (e. g. pulse, activity)
- Appointment reminders
- Appointment scheduling

34. In your opinion, is the use of a health App helpful in improving surveillance and care for individuals with a CPS, e. g. via reminders?

- Yes
- No

35. Would you recommend such an App to your patients?

- Yes
- No

36. Do you think that the Corona pandemic will lastingly change the way of dealing with digitalization in the health care system?

- Yes
- No

37. How?

- Free text

38. How do you face upcoming changes due to increasing digitalization in the health care system?

- Being hopeful
- Having mixed feelings
- Neutral
- Being worried
- Other (please specify), free text

39. Which dangers do you expect from offered online-support in the health care system? (Multiple choice)

- None
- High costs
- Data loss
- Incorrect diagnoses
- Dependency on technology

- Data abuse
- Worse doctor-patient relationship
- Other (please specify), free text

40. What changes do you expect from increasing digitalization in the following areas of healthcare?

	Improving	No change	Worsening
Availability of data	○	○	○
Access to knowledge	○	○	○
Data acquisition	○	○	○
Data quality	○	○	○
Medical research	○	○	○
Treatment of individuals with a CPS	○	○	○
Administrative medical tasks	○	○	○
Interdisciplinary collaboration	○	○	○
Participation of the patient in the documentation	○	○	○
Data transparency for patients	○	○	○
Early detection of diseases	○	○	○
Flexibility of work organization	○	○	○
Medical quality in general	○	○	○
Patient adherence	○	○	○
Popularity of the medical profession	○	○	○
Doctor-patient relationship	○	○	○

41. In your opinion, what is your role in the medical digitalization?

- Active
- Open
- Passive
- Critical
- Questioning
- Reluctant
- Other (please specify), free text
